# Biosimilar G-CSF versus filgrastim and lenograstim in healthy unrelated volunteer hematopoietic stem cell donors

**DOI:** 10.1007/s00277-017-3060-4

**Published:** 2017-08-11

**Authors:** Roiya Farhan, Elżbieta Urbanowska, Hanna Zborowska, Małgorzata Król, Maria Król, Tigran Torosian, Iwona Piotrowska, Krzysztof Bogusz, Kamila Skwierawska, Wiesław Wiktor-Jędrzejczak, Emilian Snarski

**Affiliations:** 1 Department of Hematology, Oncology and Internal Medicine, Medical University of Warsaw, Banacha 1a, Warsaw, Poland; 2 Central Laboratory, Independent Public Central Clinical Hospital, Warsaw, Poland; 3 Foundation DKMS Poland, Warsaw, Poland

**Keywords:** Biosimilar G-CSF, Filgrastim, Lenograstim, Unrelated hematopoietic stem cell donors

## Abstract

The World Marrow Donor Organization recommends original granulocyte-colony stimulating factor (G-CSF) for the mobilization of stem cells in healthy unrelated hematopoietic stem cell donors. We report the comparison of a biosimilar G-CSF (Zarzio) with two original G-CSFs (filgrastim and lenograstim) in mobilization in unrelated donors. We included data of 313 consecutive donors who were mobilized during the period from October 2014 to March 2016 at the Medical University of Warsaw. The primary endpoints of this study were the efficiency of CD34+ cell mobilization to the circulation and results of the first apheresis. The mean daily dose of G-CSF was 9.1 μg/kg for lenograstim, 9.8 μg/kg for biosimilar filgrastim, and 9.3 μg/kg for filgrastim (*p* < 0.001). The mean CD34+ cell number per microliter in the blood before the first apheresis was 111 for lenograstim, 119 for biosimilar filgrastim, and 124 for filgrastim (*p* = 0.354); the mean difference was even less significant when comparing CD34+ number per dose of G-CSF per kilogram (*p* = 0.787). Target doses of CD34+ cells were reached with one apheresis in 87% donors mobilized with lenograstim and in 93% donors mobilized with original and biosimilar filgrastim (*p* = 0.005). The mobilized apheresis outcomes (mean number of CD34+ cells/kg of donor collected during the first apheresis) was similar with lenograstim, biosimilar filgrastim, and filgrastim: 6.2 × 10^6^, 7.6 × 10^6^, and 7.3 × 10^6^, respectively, *p* = 0.06. There was no mobilization failure in any of the donors. Biosimilar G-CSF is as effective in the mobilization of hematopoietic stem cells in unrelated donors as original G-CSFs. Small and clinically irrelevant differences seen in the study can be attributed to differences in G-CSF dose and collection-related factors. Active safety surveillance concurrent to clinical use and reporting to donor outcome registry (e.g., EBMT donor outcome registry or WMDA SEAR/SPEAR) might help to evaluate the possible short- and long-term complications of biosimilar G-CSF.

## Background

There are over 27 million registered unrelated hematopoietic stem cell donors worldwide, as reported by the World Marrow Donor Association (WMDA) [1]. The standard protocol of mobilization in hematopoietic stem cell donors relies on two original granulocyte-colony stimulating factors (G-CSF): filgrastim and lenograstim. Those drugs have been evaluated in clinical trials and neither showed advantages over another [2]. However, studies conducted on healthy unrelated donors are limited, as there was only one prospective [3] and two retrospective studies comparing those drugs in this group [4, 5].

Recently, biosimilars of G-CSF have been introduced into the stem cell mobilization protocols [6]. The experience with mobilization in unrelated stem cell donors using biosimilar G-CSF is very limited [7, 8]. The WMDA and the European Society for Blood and Marrow Transplantation both argue against the use of biosimilar G-CSF [9]. On the other hand, the Working Party on Similar Biological (Biosimilar) Medicinal Products of the European Medicines Agency supports expanding the use of the biosimilars that have to provide sufficient human safety data before the approval [10].

In 2015, due to legal issues and changes in market availability, the G-CSF used in mobilization of hematopoietic stem cells in donors and patients at our institution has been first changed from lenograstim to biosimilar filgrastim and later to original filgrastim. As publications that compare biosimilars with both original G-CSFs are limited, we decided to retrospectively analyze the efficiency of mobilization with those three drugs in healthy unrelated hematopoietic stem cell donors.

## Material and methods

This study included medical data of 313 consecutive donors of both genders aged between 19 and 55 years, who were mobilized from October 2014 to March 2016. The sizes of the donor groups mobilized with original G-CSFs were chosen to resemble the size of the group mobilized with biosimilar filgrastim.

The G-CSF formulations used in this study were lenograstim—Granocyte (Chugai), biosimilar filgrastim—Zarzio (Sandoz), and filgrastim—Neupogen (Amgen). We did not report any serious adverse events (SAE) in the donors during this study (including G-CSF injections, apheresis, and postapheresis care).

Donation data were collected prospectively by our center for scientific purposes. All donors gave written, informed consent allowing the use of their anonymous medical records for research purposes. All procedures were followed in accordance with the ethical standards set by the responsible committee on human experimentation (institutional and national) and with the Helsinki Declaration of 1975, as revised in 2008. The data for this study were collected from medical records of the donors that covered qualification, collection, and, in some instances (when requested by donor center), donor follow-up. The data chosen for the study included individual donor number, type of growth factor, dose of growth factor (prior to the first apheresis and total dose), type of protocol, days of apheresis, age, sex, weight, height, dose per kilogram, dose per surface area, complete blood count at qualification, complete preapheresis blood count, percent of CD34+ cells in blood, number of CD34+ cells in blood, nucleated cell count in apheresis product/products, percent of CD34+ cells in product/products, CD34+ count (total product/products, per kg of patient weight), and complete blood count after apheresis.

### Mobilization of stem cells

The donors were sent for evaluation and collection by different donor registries. The final clearance was performed by apheresis center—the donors who were qualified had no contraindications according to the WMDA donor medical suitability recommendations. The optimal daily dose for G-CSF was selected based on the donor weight. For lenograstim, the target dose was 7.5–10 μg/kg, and for filgrastim (original and biosimilar), it was 7.5–10 μg/kg. The daily dose has been rounded to the closest possible with 34-million-IU injections of lenograstim or 30- and 48-million-IU injections of filgrastim. The G-CSF was given 4 days before the donation with the daily dose split into two injections—one given at 8 AM and the second at 8 PM. The donors were trained on how to inject G-CSF and those with no contraindications performed their injections themselves. The access to a nurse who administered injections was provided for donors who objected to self-injections.

The apheresis was performed with Spectra Optia cell separator (Terumo BCT, Lakewood, CO, USA). The separator used software version 4 up to April 20, 2015 and version 11.2 throughout the rest of the studied period. All donors were mobilized with MNC program prior to December 2015; later, cMNC was performed in 30 out of 107 donors in the original filgrastim group. Lenograstim was used until June 2015, biosimilar filgrastim from June to October 2015, and filgrastim was used afterwards. ACD-A was used as coagulation in a proportion of 0.9 AC.

The donors had one or two aphereses as needed to collect the number of the CD34+ cells required by the transplant center. The CD34+ cell count has been evaluated according to the ISHAGE guidelines (dual-platform method) [11]. Statistical analysis was performed with MedCalc Statistical Software version 15.10 (MedCalc Software BVBA, Ostend, Belgium). In all analyses, a *p* value of <0.05 was considered statistically significant. The center evaluates CD34+ methodology by performing CD34+ enumeration 6–8 times a year with a BD Stem Cell Control Kit (BD Bioscences, San Jose, USA). There were no reports of significant discrepancies in CD34+ cell count from transplant centers in the studied period.

If one apheresis provided over 95% of requested stem cells, the second apheresis was not performed. In the case of a second apheresis, the time of the procedure (and the volume of the product) was reduced in selected cases, so as not to mobilize with excess. Mobilization failure was defined as collection of less than 2 × 10^6^ of CD34+ cells per kilogram of body weight of the recipient.

The primary endpoints of the study were the efficiency of CD34+ cell mobilization to the circulation (measured as the number of CD34+ cells per microliter prior to the initiation of the first apheresis) and results of the first apheresis. The study was not designed to analyze short- and long-term complications of G-CSF.

## Results

Altogether, 313 consecutive healthy donors were included in this study. One hundred twenty-one received lenograstim, 85 biosimilar filgrastim, and 107 filgrastim as G-CSF in the mobilization of hematopoietic stem cells. All three groups had similar demographic data regarding sex distribution and median age; however, the group mobilized with biosimilar G-CSF had higher body mass (+11% when compared to the median weight in two other groups of patients, *p* = 0.02), and the group mobilized with filgrastim had higher body mass index than the other two groups, 25.6 vs 24.5 and 24.2 (*p* = 0.018) (Table 1).

**Table 1:** Basic data of the study population of unrelated donors

	Lenograstim	Biosimilar filgrastim	Filgrastim	*p* value
Number of donors in the group	121	85	107	
Median age (interquartile range, years)	29 (23–34)	26 (22–33)	29 (24–35.5)	0.251
BMI (interquartile range)	24.5 (22.1–26.7)	24.2 (21.5–26.2)	25.6 (22.7–28.4)	0.018
Gender	71 males/50 females	60 males/25 females	70males/37 females	0.107
Median weight of the donor (interquartile range, kg)	74 (63.5–85)	82 (63.5–85)	74 (69–90)	0.0262
Mean daily dose of G-CSF (interquartile range, μg/kg of donor)	9.1 (8.3–9.9)	9.8 (9.0–10.4)	9.3 (8.7–10.0)	<0.001

The mean daily dose of G-CSF was 9.1 μg/kg for lenograstim, 9.8 μg/kg for biosimilar filgrastim, and 9.3 μg/kg for filgrastim (*p* < 0.001). The total mean dosage of G-CSF to the first apheresis was different in all groups: 40.7 μg/kg for lenograstim (29.0–52.4), 44 μg/kg for biosimilar filgrastim (36–60), and 42 μg/kg for filgrastim (33–55)—the donors who were mobilized with biosimilar filgrastim received mean higher total doses of G-CSF (*p* = 0.014 vs filgrastim, *p* < 0.001 vs lenograstim). After four doses of G-CSF but before the last dose of G-CSF prior to apheresis, the mean white blood cell count was 43.7 g/l for lenograstim (33.8–52.5 interquartile range), 45.2 g/l for biosimilar filgrastim (36.9–52.2 interquartile range (IQR)), and 46.4 g/l for filgrastim (37.3–55.4 IQR) (*p* = 0.246). The mean CD34+ cell number in the blood before the initiation of apheresis was 111 cells/μl for lenograstim (63–146 IQR), 119 cells/μl for biosimilar filgrastim (83–157 IQR), and 124 cells/μl for filgrastim (73–162 IQR) (*p* = 0.354).

The mobilization with filgrastim and biosimilar filgrastim was more efficient than that with lenograstim: 93% of donors after one of the filgrastims needed one apheresis for sufficient collection compared to 87% donors after lenograstim (*p* = 0.005). The mean number of collected CD34+ cells × 10^6^ per kilogram of donor: 6.2 for lenograstim, 7.6 for biosimilar filgrastim, and 7.3 for filgrastim (*p* = 0.06). After correcting for donor gender, the mean number of collected CD34 + cells × 10^6^ per kilogram of recipient did not differ between the groups (*p* = 0.129) but generally higher collections were reported in men than in women (9.44 vs 6.3, *p* < 0.0001). Additionally, gender had the same effect on collection with all three growth factors (*p* = 0.847). There were no differences regarding the number of collected CD34+ cells × 10^6^ per kilogram of recipient between donors when MNC or cMNC was used (7.5 vs 8.6, *p* = 0.334) and those who underwent aphereses using different versions of cell separator software (*p* = 0.086). There were no mobilization failures, with 100% of donors mobilizing over 2 × 10^6^ CD34+ cells/kg of recipient. The mean difference in the total number of collected CD34+ cells × 10^6^ per kilogram of recipient between different G-CSFs was also not statically significant: 7.7 for lenograstim, 8.5 for biosimilar filgrastim, and 9.4 for filgrastim (*p* = 0.085). The most important comparisons between mobilization with lenograstim, biosimilar filgrastim, and filgrastim are summarized in Table 2.

**Table 2:** The most important differences between G-CSFs in mobilization of unrelated hematopoietic stem cells donors

	Lenograstim	Biosimilar filgrastim	Filgrastim	*p* value
Mean preapheresis white blood cell count (interquartile range, g/l)	43.7 (33.8–52.5)	45.2 (36.9–52.2)	46.4 (37.3–55.4)	0.246
Mean preapheresis erythrocytes count (interquartile range, g/l)	4.70 (4.38–4.95)	4.76 (4.44–5.09)	4.79 (4.55–5.06)	0.193
Mean preapheresis thrombocytes count (interquartile range, g/l)	237 (198–267)	236 (196–276)	250 (215–285)	0.126
Mean preapheresis CD34+ count (interquartile range, cells/μl)	111 (63–136)	119 (83–157)	124 (73–162)	0.354
Mean number of CD34+ cells collected from first apheresis (interquartile range, ×10^6^/kg of recipient)	7.5 (4.5–9.2)	8.3 (5.2–9.8)	9.4 (5.5–11.0)	0.06
Mean number of CD34+ cells collected from all aphereses^a^ (interquartile range, ×10^6^/kg of recipient)	7.7 (4.7–9.2)	8.5 (5.7–9.8)	9.4 (5.5–11.0)	0.085
Growth factor efficiency (interquartile range, mean number of CD34+ cells from first apheresis per kg per daily G-CSF dose in μg/kg)	0.68 (0.42–0.87)	0.75 (0.52–0.95)	0.77 (0.57–0.97)	0.074
Percentage of donors that needed one apheresis for collection (%)	87	93	93	0.005
Mean postapheresis white blood cell count (interquartile range, g/l)	40.1 (32.4–46.1)	46.2 (39.5–51.0)	46.24 (38.0–55.6)	<0.001
Mean postapheresis erythrocytes count (interquartile range, g/l)	4.41 (4.05–4.70)	4.52 (4.18–4.83)	4.58 (4.28–4.84)	0.013
Mean postapheresis thrombocytes count (interquartile range, g/l)	147 (118–169)	152 (122–178)	167 (138–193)	<0.001

^a^All aphereses—one or maximum two aphereses in a donor

## Discussion

The biosimilar G-CSF is not used as standard in the mobilization of hematopoietic stem cells in unrelated donors. Due to the unlikely coincidence of issues with the availability of the drugs on the market, we were able to retrospectively compare the two original G-CSFs (lenograstim and filgrastim) with biosimilar filgrastim. The primary endpoints of our study, mobilization of CD34+ into circulation and results of the first apheresis, have shown no statistically significant differences between original and biosimilar drugs. We observed almost no clinically relevant difference between the drugs—the number of donors needing one apheresis is similar in three groups—with differences related possibly to slight differences in dose, sex distribution, and procedure of cell separation.

Previous studies have also shown differences between lenograstim and filgrastim—however, in favor of lenograstim. Ings et al. [5] have shown that lenograstim had higher CD34+ cell collections—but they have failed to show data on actual G-CSF doses in studied groups and their groups had different sex distributions. Higher efficiency of lenograstim was also reported by Bertani and colleagues [12]. The data in both of those studies could also be influenced by the use of different cell separators and their software, as it took 10 [5] or 18 [12] years to collect patient data. Similar results were also reported in a prospective study by Fischer and colleagues [3]—again, reporting roughly 10% higher stem cell collection yields using lenograstim, with very similar fist apheresis collection numbers to those reported in this publication. The actual dose of G-CSF in that study was almost 20% higher than that applied to the patients in our analysis—most likely influencing the observed differences. In our study, a small but statistically significant dose differences in lenograstim and filgrastims could have influenced the final CD34+ cell yield. The results of this study show that biosimilar filgrastim performs almost identically to original products. The ongoing prospective study will provide more data on this topic—most importantly assessing the differences in side effects between the drugs [8].

The biggest limitation of this study was the inability to assess the short- and long-term side effects of the studied drugs. On the other hand, the short-term side effects of the drugs will likely not differ significantly [8]. We also do not report cell separation data and only focus on preapheresis data and product data. There is a possibility that the outcomes could have been influenced by differences in cell separation which is beyond the scope of the study.

## Conclusion

Our data provide positive evidence for the use of biosimilar G-CSF in healthy unrelated hematopoietic stem cell donors. It is shown that the biosimilar G-CSF does not differ from the original growth factors when the mobilization of the CD34+ positive cells is analyzed. We still need to wait for the results of long-term observations of the donors after biosimilar G-CSF before we can recommend its use as standard in the mobilization of hematopoietic stem cells in healthy unrelated donors.
